# Avian Influenza Virus H3 Hemagglutinin May Enable High Fitness of Novel Human Virus Reassortants

**DOI:** 10.1371/journal.pone.0079165

**Published:** 2013-11-12

**Authors:** Anne Kreibich, Olga Stech, Jana Hundt, Mario Ziller, Thomas C. Mettenleiter, Juergen Stech

**Affiliations:** 1 Friedrich-Loeffler-Institut, Institute of Molecular Biology, Greifswald-Insel Riems, Germany; 2 Friedrich-Loeffler-Institut, Biomathematics Working Group, Greifswald-Insel Riems, Germany; University of Edinburgh, United Kingdom

## Abstract

Reassortment of influenza A virus genes enables antigenic shift resulting in the emergence of pandemic viruses with novel hemagglutinins (HA) acquired from avian strains. Here, we investigated whether historic and contemporary avian strains with different replication capacity in human cells can donate their hemagglutinin to a pandemic human virus. We performed double-infections with two avian H3 strains as HA donors and a human acceptor strain, and determined gene compositions and replication of HA reassortants in mammalian cells. To enforce selection for the avian virus HA, we generated a strictly elastase-dependent HA cleavage site mutant from A/Hong Kong/1/68 (H3N2) (Hk68-Ela). This mutant was used for co-infections of human cells with A/Duck/Ukraine/1/63 (H3N8) (DkUkr63) or the more recent A/Mallard/Germany/Wv64-67/05 (H3N2) (MallGer05) in the absence of elastase but presence of trypsin. Among 21 plaques analyzed from each assay, we found 12 HA reassortants with DkUkr63 (4 genotypes) and 14 with MallGer05 (10 genotypes) that replicated in human cells comparable to the parental human virus. Although DkUkr63 replicated in mammalian cells at a reduced level compared to MallGer05 and Hk68, it transmitted its HA to the human virus, indicating that lower replication efficiency of an avian virus in a mammalian host may not constrain the emergence of viable HA reassortants. The finding that HA and HA/NA reassortants replicated efficiently like the human virus suggests that further HA adaptation remains a relevant barrier for emergence of novel HA reassortants.

## Introduction

Several influenza pandemics in the last century were caused by influenza A viruses carrying a hemagglutinin (HA) serotype novel to the global human population. Repeated emergence of those heterologous strains was facilitated by the segmentation of the viral genome into eight genes. Such a genome organization allows the exchange of entire gene segments during co-infection by two different strains, designated as reassortment [1]. Besides the hemagglutinin-encoding segment, other genes of the prevailing human strain were also replaced by gene segments derived from an unknown avian strain. The 1957 pandemic was caused by a reassortant in which the genes encoding HA, neuraminidase (NA) and polymerase basic protein 1 (PB1) of the prevalent human H1N1 strain were replaced by those from an avian H2N2 strain. In the subsequent 1968 pandemic, another reassortant emerged carrying HA and PB1 genes from an avian H3 strain [2]–[5]. In contrast, the 2009 pandemic was caused by an H1 swine virus reassortant exhibiting minor antigenic shift of HA and NA [6]–[8]. Since documented human pandemics were caused by H1, H2 and H3 strains, low-pathogenic avian or mammalian field strains specifying those HA serotypes continue to deserve particular attention since they may serve as potential HA donors for novel pandemic strains. Low-pathogenic avian influenza viruses (LPAIV) are frequent in wild aquatic birds, especially in mallards, at isolation rates up to 11% with the HA serotypes H1, H2, and H3 amounting to a portion of approximately 8% [9], [10]. However, the compatibility of the HA gene of those avian H1, H2 or H3 strains with the other genes of established human strains is unclear. In this study, we investigated the ability of two avian H3 LPAIV to give rise to efficiently replicating HA reassortants with a human pandemic H3 virus. We analyzed whether the replication efficiency of the novel reassortants was comparable to that of the human parent virus and determined which gene segments co-segregated with the HA gene. To enforce selection for avian virus HA reassortants, we generated a strictly elastase-dependent HA cleavage-site mutant from A/Hong Kong/1/68 (H3N2) (Hk68) to serve as human acceptor virus, and performed co-infections of human cells with an LPAIV in the absence of elastase but presence of trypsin. As HA donors, we used A/Duck/Ukraine/1/63 (H3N8) (DkUkr63), which is related to the unknown progenitor of the pandemic virus Hk68 [11] but impaired in replication in human cells, and the more recent isolate A/Mallard/Germany/Wv64-67/05 (H3N2) (MallGer05) which replicates well in cells of human origin.

## Results

### Generation of the Elastase-dependent HA Cleavage Site Mutant Hk68-Ela for use as Acceptor Virus

To enforce selection of the avian virus HA during co-infection in the absence of elastase, we generated an elastase-dependent HA cleavage site mutant (HK68-Ela) from the human influenza strain Hk68 [12], [13]. First, we replaced the HA cleavage site residues P4 to P1 by four alanine residues (amino acid positions 342–345 corresponding to nucleotides 1053–1067) resulting in an elastase motif [14], [15]. Then, the modified HA plasmid was co-transfected with the other seven gene segments of Hk68 (PB2, PB1, PA, NP, NA, M, and NS genes) [13] to obtain Hk68-Ela. Its strict dependence on elastase for multi-cycle replication was demonstrated by the absence of plaque formation in the presence of trypsin or in the absence of elastase (Figure S1).

### Enforced Selection of Reassortants Carrying the Avian Virus HA

**Figure 1:**
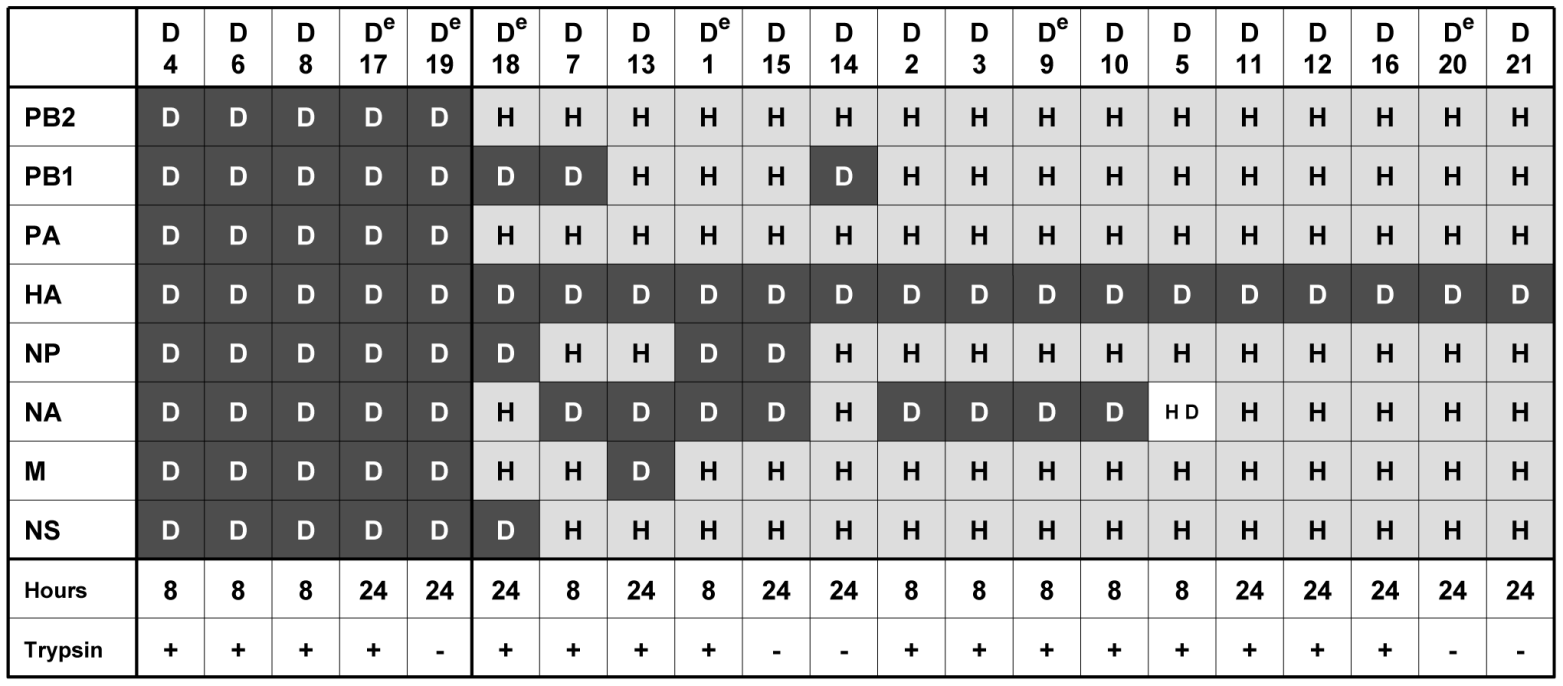
Gene composition of DkUkr63 reassortants. Gene segments originating from DkUkr63 (D) and Hk68 (H) are shown in dark and light grey, respectively (HD indicates a mixed population). Co-infections with Hk68-Ela and DkUkr63 were performed either in the presence (+) or absence (−) of TPCK-treated trypsin. Supernatants were harvested either at eight or 24 hours after co-infection, the RNA isolated and subjected to RT-PCR for genotyping. Reassortants with no detectable HA titer in the supernatant after co-infection were propagated in embryonated chicken eggs and are indicated by a super-scripted ^e^.

To select reassortants carrying the HA of an avian strain, we performed co-infection experiments on human A549 cells in the absence of elastase using Hk68-Ela as acceptor strain and either DkUkr63 (H3N8) or the more recent MallGer05 (H3N2) as HA donor. After co-infection, we harvested supernatants from infected cells after 8 and 24 hours and performed plaque assays. From each co-infection experiment, we isolated 21 plaques of different sizes and passaged them on A549 cells in the presence or absence of trypsin. If no HA titer was detectable in the supernatant, we added an egg passage. From those propagated virus stocks, we determined partial sequences of each segment for assigning the parental origin. For the DkUkr63 co-infection, genotyping revealed that 16 of 21 (D1–D21) single plaque isolates were reassortants carrying the avian HA either alone or in combination with other DkUkr63 genes (Figure 1). NA co-segregated with the avian HA in approximately half of the reassortants. In three reassortants each, PB1 (D7, D14, D18) and NP genes (D1, D15, D18) originated from DkUkr63. The M and NS genes were replaced in only one reassortant each (D13 and D18, respectively). PB2 and PA never co-segregated with the DkUkr63 HA. Only one of the 16 reassortants (D18) incorporated more than three segments of DkUkr63 origin. Remarkably, the reassortant D7, carrying the DkUkr63 PB1, HA and NA segments, exhibited a gene constellation similar to that of the 1957 pandemic virus (Figure 1).

**Figure 2:**
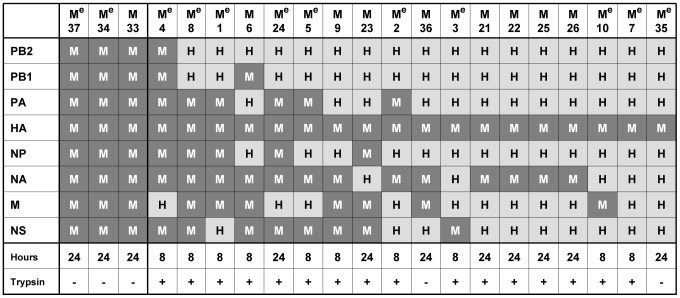
Gene composition of MallGer05 reassortants. Gene segments originating from MallGer05 (M) and Hk68 (H) are shown in dark and light grey, respectively. Co-infections with Hk68-Ela and MallGer05 were performed either in the presence (+) or absence (−) of TPCK-treated trypsin. Supernatants were harvested either at eight or 24 hours after co-infection, the RNA isolated and subjected to RT-PCR for genotyping. Reassortants with no detectable HA titer in the supernatant after co-infection were propagated in embryonated chicken eggs and are indicated by a super-scripted ^e^.

The second co-infection experiment with the more recent avian strain MallGer05 yielded 18 HA reassortants of the 21 genotyped plaque isolates (M1–M21) (Figure 2). NA co-segregated with HA in 13 of the reassortants. Eight of the 18 reassortants incorporated more than three MallGer05 segments, i.e. the PA, NP, M, and NS genes; only two reassortants carried the PB1 and one the PB2 from MallGer05. Taken together, both avian strains readily formed HA reassortants. However, whereas only 1 of the 16 DkUkr63 reassortants (D18) carried three avian virus segments in addition to the HA gene, eight of 18 MallGer05 reassortants acquired three or more avian virus gene segments in addition to the HA gene.

### Plaque Morphology of Reassortants

The plaque morphology of parental and reassortant viruses on MDCK cells differs considerably. Whereas Hk68 and Hk68-Ela yielded pinpoint plaques, DkUkr63 and MallGer05 formed large, clear plaques of approximately 4.0 mm and 2.5 mm in diameter, respectively. Reassortants carrying the N2 from Hk68 (for example D12, D14, D18 and M3, M7, M23) displayed tiny, hardly visible plaques like the human parent. In contrast, reassortants carrying the N8 from DkUkr63 (as D7, D9, and D13) or the N2 from MallGer05 (as M4, M6, and M26) formed large plaques comparable to those of their respective avian parental virus (Figure S2). Taken together, the plaque morphology of the reassortant viruses depends on the origin of the NA corresponding to previous findings [16].

### Replication Efficiency of DkUkr63 Reassortants in Mammalian Cells

**Figure 3:**
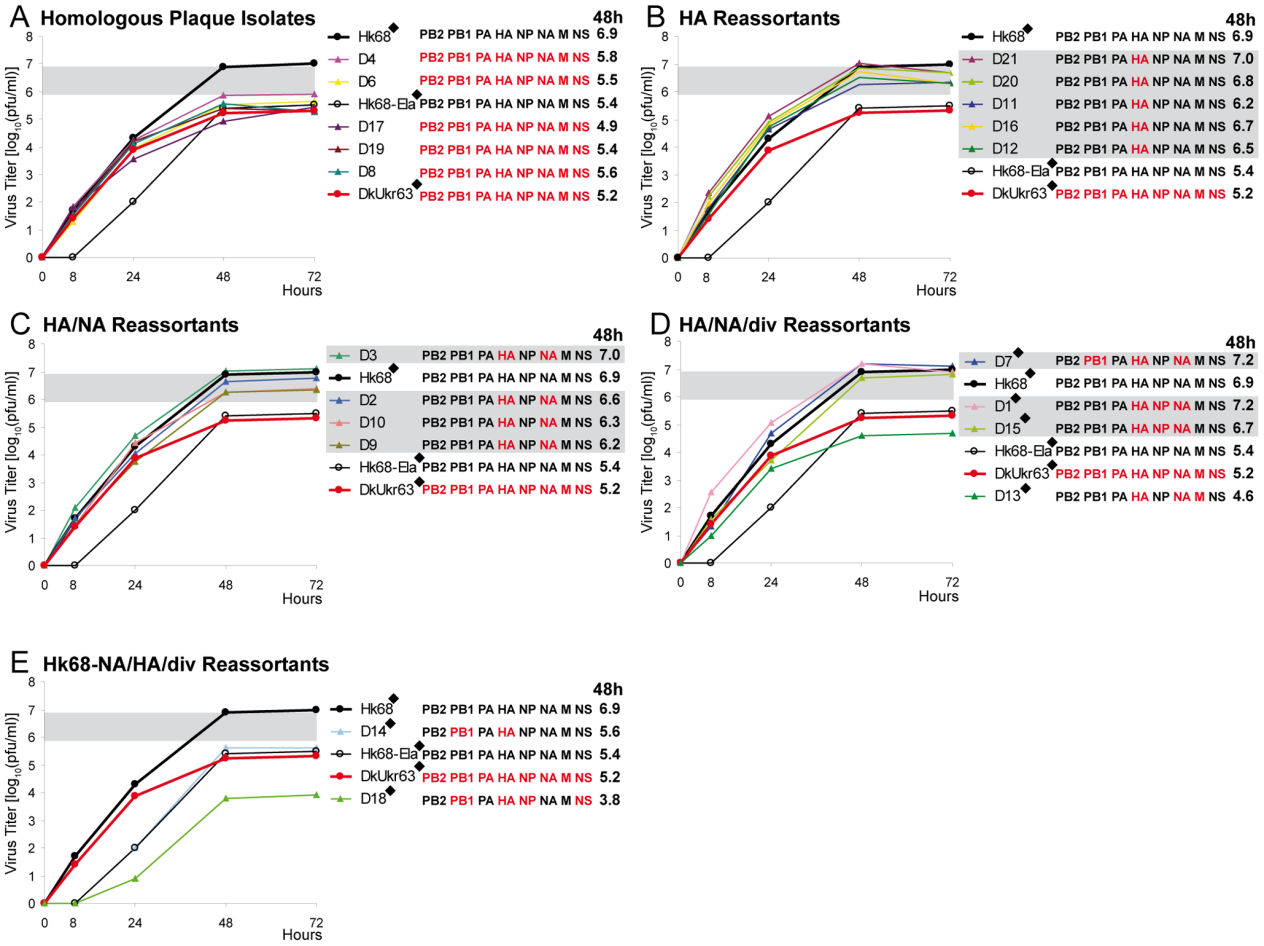
Growth curves of DkUkr63 HA reassortants. The gene segment constellation of each reassortant is given next to each chart: Hk68 genes (black) and DkUkr63 genes (red). The presence of diverse additional DkUkr63 segments is indicated by the abbreviation ‘div’. From supernatants of A549 cells infected with a MOI of 10^−3^, viral titers were determined by plaque assays on MDCK cells. The growth curves of the parental viruses Hk68 (black filled circles), Hk68-Ela (black empty circles) and DkUkr63 (red filled circles) in each chart represent identical data and were drawn in for comparison. Growth curves were determined by plaque titrations of duplicate or quadruplicate infected cell cultures (in the latter case indicated by a diamond in the chart legend). Reassortants reaching titers at 48 h not less than one magnitude below Hk68 are highlighted by grey rectangles.

The parental Hk68 replicated in A549 cells to a titer of 6.9 log_10_(pfu/ml) after 48 h. The elastase cleavage site mutant Hk68-Ela replicated with a delay and reached a titer of 5.4 log_10_(pfu/ml) at 48 h in the presence of elastase. DkUkr63 replicated as efficiently as Hk68 during the first 24 hours but reached a lower titer of 5.2 log_10_(pfu/ml) after 48 h (Figure 3A). According to their genotypes, the plaques-isolates obtained after co-infection can be divided into five groups: (1) Five plaque-isolates represented parental DkUkr63 (D4, D6, D17, D19, and D8) yielding growth curves similar to DkUkr63 (Figure 3A, Table S1). (2) Five reassortants contained only the HA of DkUkr63 (D21, D20, D11, D16, and D12): Their 48 h titers ranged between 6.2 and 7.0 log_10_(pfu/ml) (Figure 3B, Table S1) corresponding to an increase of approximately 10-fold compared to DkUkr63 (5.2 log_10_(pfu/ml)). (3) Four reassortants carrying HA and NA from DkUkr63 (D3, D2, D10, and D9) also replicated more efficiently than DkUkr63 reaching 48 h titers between 6.2 and 7.0 log_10_(pfu/ml) (Figure 3C, Table S1). (4) Four reassortants carried HA and NA plus one or more additional diverse segments from DkUkr63. One of the best replicating reassortants was D7, containing the DkUkr63 HA, NA and PB1, a gene constellation similar to that of the 1957 Asian pandemic virus. D7 reached a 48 h titer of 7.2 log_10_(pfu/ml), slightly higher than that of Hk68 (6.9 log_10_(pfu/ml)). Similar titers were observed for D1 and D15 carrying DkUkr63 HA, NA and NP (7.2 and 6.7 log_10_(pfu/ml)). In contrast, reassortant D13 carrying the DkUkr63 M gene in combination with DkUkr63 HA and NA genes, exhibited significantly reduced replication with a 48 h titer of 4.6 log_10_(pfu/ml) that is even below that of the parent virus DkUkr63 (final titer 5.2 log_10_(pfu/ml)) (Figure 3D, Table S1). (5) Two reassortants containing the DkUkr63 HA but the Hk68 NA showed a delayed growth already at 8 and 24 h in comparison to the parental viruses. Reassortant D14 with DkUkr63 HA and PB1 replicated to 5.6 log_10_(pfu/ml), and reassortant D18 with DkUkr63 HA, NA, PB1, and NS showed the strongest, significant growth impairment with a 48 h titer of 3.8 log_10_(pfu/ml) (Figure 3E, Table S1).

Taken together, most DkUkr63 HA reassortants reached titers in A549 cells higher than the parent virus DkUkr63. The titers of all HA or HA/NA reassortants deviated only up to one magnitude from that of Hk68 demonstrating that donation of DkUkr63 HA and NA genes to a human strain resulted in reassortants with robust replication competence in A549 cells. Highest replication efficiency was observed with reassortants D1 and D15 containing the DkUkr63 HA, NA and NP, and in particular with reassortant D7 carrying the DkUkr63 HA, NA and PB1 and, thus, specifying a similar gene constellation as that of the 1957 pandemic strain.

### Replication Efficiency of MallGer05 Reassortants in Mammalian Cells

**Figure 4:**
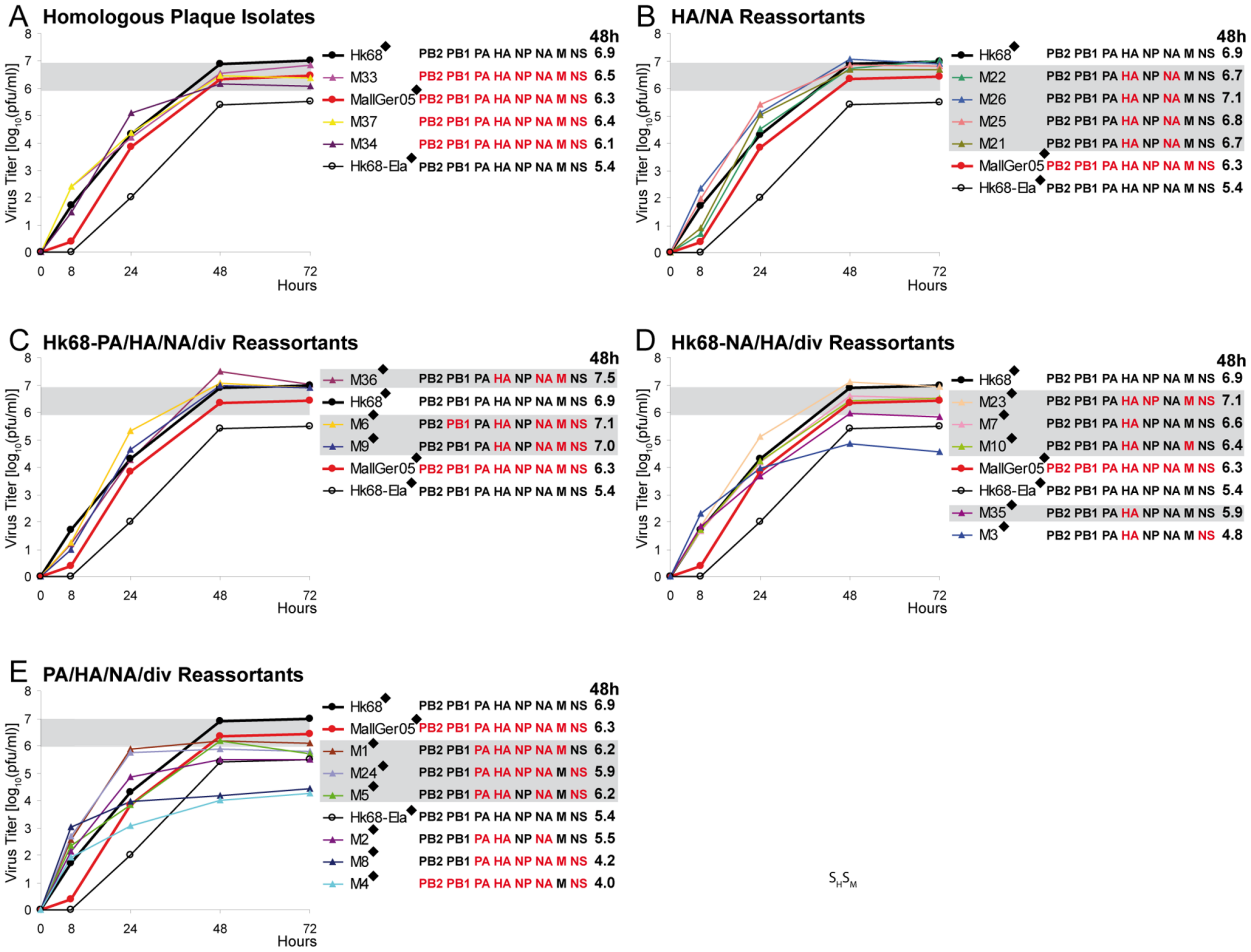
Growth curves of MallGer05 HA reassortants. The gene segment composition of the each reassortant is given next to each chart: Hk68 genes (black) and MallGer05 genes (red). The presence of diverse additional MallGer05 segments is indicated by the abbreviation ‘div’. From supernatants of A549 cells infected with a MOI of 10^−3^, viral titers were determined by plaque assays on MDCK cells. The growth curves of the parental viruses Hk68 (black filled circles), Hk68-Ela (black empty circles) and MallGer05 (red filled circles) in each chart represent identical data and were drawn in for comparison. Growth curves were determined by plaque titrations of duplicate or quadruplicate infected cell cultures (in the latter case indicated by a diamond in the chart legend). Reassortants reaching titers at 48 h not less than one magnitude below Hk68 are highlighted by grey rectangles.

The parental MallGer05 reached a 48 h titer of 6.3 log_10_(pfu/ml) in A549 cells which was close to that of Hk68 with 6.9 log_10_(pfu/ml) (Figure 4A). According to their gene constellations, the single plaque isolates obtained after co-infection were divided into five groups: (1) Three plaque-isolates represented parental MallGer05 (M33, M37, and M34) and reached similar 48 h titers as MallGer05 (Figure 4A, Table S2). (2) Four reassortants carried only the HA and NA genes from MallGer05 (M21, M26, M25, M21). They reached 48 h titers between 6.7 to 7.1 log_10_(pfu/ml) similar to Hk68 with 6.9 log_10_(pfu/ml) (Figure 4B, Table S2). (3) Three reassortants carried HA, NA plus one or two other genes from MallGer05, in combination with Hk68 PA. These reassortants reach 48 h titers equivalent to Hk68: M36 (MallGer05 HA, NA, and M) 7.5 log_10_(pfu/ml), M6 (MallGer05 HA, PB1, NA, M, and NS) 7.1 log_10_(pfu/ml), and M9 6.9 (MallGer05 HA, NA, M, and NS) log_10_(pfu/ml) (Figure 4C, Table S2). (4) Five reassortants carried the MallGer05 HA and several other MallGer05 genes combined with the NA from Hk68. Reassortant M23 (MallGer05 HA, NP, M and NS) reached a 48 h titer of 7.1 log_10_(pfu/ml) equivalent to Hk68. M7 and M35 (only MallGer05 HA) and M10 (MallGer05 HA and M) replicated to 48 h titers comparable to MallGer05. M3 (MallGer05 HA and NS) exhibited a 48h titer of 4.8 log_10_(pfu/ml) which was approximately 100-fold below Hk68 (Figure 4D, Table S2). (5) Six reassortants carried MallGer05 PA combined with other MallGer05 genes. All those viruses except M4 displayed enhanced early replication compared with Hk68, but did not reach equivalent titers. M1 (MallGer05 PA, HA, NP, NA, and M), M24 (MallGer05 PA, HA, NP, NA, and NS), and M2 (MallGer05 PA, HA, and NA) reached higher titers than Hk68 at 24 hours but eventually stagnated at 48 h titers between 5.9 and 6.2 log_10_(pfu/ml). Remarkably, M8 (only PB2 and PB1 from Hk68) and M4 (only M from Hk68) reached final titers of only 4.2 and 4.0 log_10_(pfu/ml), respectively, demonstrating severely impaired, significant replication (Figure 4E, Table S2).

In sharp contrast to the DkUkr63 M gene, the MallGer05 M gene did not restrict host range since the HA/NA/M (M36), PB1/HA/NA/M/NS (M6), and HA/NA/M/NS (M9) reassortants replicated better than Hk68. Most notably, all HA/NA reassortants (4 of 18) reached 48 h titers very close to that of Hk68 indicating the ability of MallGer05 to donate its HA and NA genes efficiently to a human strain.

## Discussion

In the past century, several pandemics were caused by influenza A strains that had reassorted their HA genes. This enormous genetic plasticity results from the segmentation of their viral genomes enabling reassortment. Replacement of the HA gene segment in a seasonal human strain by that from an avian strain has led to the emergence of pandemic strains with novel HA serotypes in 1957 and 1968 [2]–[5]. Thus, such a reassortment event still has to be considered a relevant scenario. In this study, we analyzed whether an early and a more recent H3 avian strain which differ in their replication capacity in human cells, may form viable HA reassortants with a human strain. Furthermore, we identified the gene segments which co-segregated with the HA gene. To this end, we performed co-infections with the elastase-dependent HA cleavage site mutant Hk68-Ela of the pandemic influenza virus Hk68 (H3N2) and either the avian DkUkr63 (H3N8) or the more recent MallGer05 (H3N2) in the absence of elastase but in the presence of trypsin. DkUkr63 and MallGer05 had before been passaged in eggs only five or two times, respectively, which renders artifacts due to egg-adaptation unlikely. Since low-pathogenic avian viruses depend on trypsin-like proteases for multicycle replication [17], the strict elastase dependency of the acceptor virus Hk68-Ela allowed us to enforce selection of reassortants carrying the avian virus HA either alone or in combination with other avian virus gene segments. With a strictly elastase-dependent acceptor virus, the occurrence of escape mutants is less likely than by antibody selection [18] since two concurrent point mutations at the HA cleavage site are required for reversion to a monobasic motif [12]. Furthermore, the use of an elastase-dependent mutant as acceptor virus facilitated the selection of HA reassortants even if both parental viruses share the same HA serotype. The higher frequency of Hk68 genes observed among the reassortants might be due to the 100fold higher inoculation dose of Hk68-Ela compared to both avian viruses. Overall, both, DkUkr63 and MallGer05 transmitted their HA to the human virus Hk68 either alone or in combination with other gene segments, resulting in several HA reassortants with a replication capacity in human cells comparable to the human parental virus.

To reveal whether some gene constellations of reassortants were restricted, we assumed the absence of any restriction and considered the distribution of genotypes in relation to the number of isolated plaques to be a coupon collector problem [19], [20]. Due to enforced selection of the avian virus HA during co-infection, 126 different reassortant genotypes (2^7^ possible unique combinations of viral genes minus those of the two homologous parent viruses) are to be expected at equal frequencies among sufficiently high numbers of isolated plaques in case of the absence of any restriction against the other seven virus genes. Among 21 investigated plaques for each co-infection, we found with DkUkr63 16 and with MallGer05 18 reassortants. We anticipated the occurrence of approximately 15 different genotypes (i.e. unique gene combinations) for DkUkr63 and 17 different genotypes for MallGer05 according to a martingale-based exact formula of the coupon collection problem [19], [20] and Monte Carlo simulations [21]. However, the genotyping revealed 7 and 14 different unique gene combinations, respectively, suggesting a more stringent restriction against DkUkr63 genes than MallGer05 genes. The gene constellations found are independent from a subsequent passage in embryonated eggs after infection of A549 cells. Nevertheless, DkUkr63 yielded four and MallGer05 10 different genotypes with growth properties comparable to the human strain Hk68 (Figure 3 and 4, Table S3); the close differences in growth titers of those genotypes to Hk68 are mostly not significant (Tables S1 and S2). Because statistical testing estimates the significance of notable titer differences, non-significance as found for the HA reassortants vs. the human and the respective avian parent virus, indicates absence of evidence but not evidence of absence of a notable difference, i.e. equivalence. Remarkably, in contrast to MallGer05, the replication of DkUkr63 in human A549 cells was severely impaired compared with Hk68. Accordingly, the formation of reassortants from both avian donor viruses irrespective the presence of trypsin during co-infection (Figure 1 and 2) suggests that reassortment may occur already during the first replication cycle. Therefore, novel efficiently replicating HA reassortants might emerge from LPAIV serving as HA donors even if they are poorly adapted to the human host.

Pinpoint plaques similar to those formed by Hk68 parental virus were observed for reassortants carrying an Hk68 NA as for D12 or M7 which had acquired only the avian virus HA. In contrast, the DkUkr63 and MallGer05 HA/NA double reassortants, as D9 and M26, yielded plaque sizes comparable to those of the respective avian parent virus. Thus, irrespective of the origin of the HA and the presence of any other avian virus genes, the plaque size was determined by the NA corresponding to previous findings [16].

Efficiently replicating reassortants with heterologous polymerase complexes containing the DkUkr63 PB2 or PA were not isolated. In case of the MallGer05 reassortants, all six PA reassortants (Figure 4E) exhibited increased early virus replication but considerably varying final titers. It appears that the presence of the Hk68 NP, M or NS compensates this deficit to some extent. On the other hand, reassortment has been shown to be restricted by incompatibilities between the polymerase proteins [22]. Likewise, the isolated transfer of the PB2 gene resulted in severe replication defects [23]. Therefore, our results suggest that efficiently replicating P gene reassortants had been present after double-infection but could not be found among the 21 isolated plaques or there is a restriction against the P genes from avian viruses. Such a restriction might come into effect in a mammalian host (as the A549 cells used for double-infection) due to the absence of adaptive mutations like PB2 E627K or D701N [24]–[26] both in DkUkr63 and MallGer05 (data not shown).

In contrast to the PB2 and PA genes, the PB1 gene was found in several reassortants with different replication efficiencies. One DkUkr63 reassortant with exchanged PB1, HA, and NA achieved highest titers (D7), whereas a PB1/HA reassortant displayed impaired growth (D14). One MallGer05 reassortant carrying the PB1, HA, NA plus the M and NS genes replicated efficiently in the human cells. This finding is consistent with the acquisition of the PB1 gene of the 1957 and 1968 pandemic viruses from an unknown avian strain [5]. Our observation and the repeated occurrence of well-replicating PB1 reassortants [22], [23] could be attributed to a lower restriction against PB1 in contrast to PB2 and PA [22], [23], [27].

The M gene was shown to segregate with the HA in two HPAIV [28], [29]. On the other hand, there may be a strain-specific cooperation of the M gene from a human virus with avian virus HA [18]. Here, we found only one DkUkr63 M reassortant (D13, HA/NA/M) which displayed considerably impaired growth in contrast to DkUkr63 HA and HA/NA reassortants which had retained the Hk68 M gene. With MallGer05, all HA and HA/NA reassortants grew comparable to Hk68 irrespective of the origin of the M gene. These observations indicate that the M gene may not generally segregate with HA. Furthermore, the single M gene reassortant M4 containing the Hk68 M gene in the MallGer05 background, exhibited severely impaired replication. However, the other Hk68 M gene reassortants M24, M5 and M2 reached considerably higher titers indicating compensation of the defect by the presence of Hk68 PB2 and PB1 plus either NP or NS. Thus, we hypothesize that lower fitness of one gene can be overcome by other gene segments due to better adaptation or co-packaging.

For virus entry, human strains use surface glycans with α(2,6)-linked sialic acids as receptors, whereas avian strains prefer α(2,3)-linked sialic acids [30], [31]. For selection of HA reassortants, we used A549 cells which carry α(2,3)- as well as α(2,6)-linked sialic acids [32]. This feature allowed us to investigate the ability of an avian virus HA to appear in the genetic background of a human strain per se because in those cells, virus receptor distribution does not act as host barrier. However, in mammalian hosts such as pigs and ferrets, the α(2,6)-linked receptor preference, besides an enhanced activity of the viral polymerase, is essential for full adaptation of H3 and H5 strains in terms of efficient replication in the upper respiratory tract enabling airborne transmission [33]–[39]. Correspondingly, for the pandemic 1918 H1N1 strain, the exchange of two amino acids determining the receptor-binding specificity of the HA prevented airborne transmission among ferrets [40]. On the other hand, two recent ferret adaptation studies on airborne transmissibility of a mutated H5N1 strain and an H5 reassortant derived from a pandemic 2009 virus revealed a requirement for a minimum of four HA mutations. These identified substitutions affect the receptor binding site (RBS), the trimer interface and protein stability [38], [39]. Whereas those additional HA mutations had been identified in naturally occurring HPAIV [38], [39], [41], the adaptation of the RBS to the human host already in waterfowl is unlikely because in mallards HA mutants with human-like receptor specificity are severely restricted or are even forced to reversion [42], [43]. Since LPAIV can be isolated from humans with underlying conditions such as an H7N2 low-pathogenic strain from an immunocompromised patient [44] and reassortment may take place within one replication cycle, it remains conceivable that the final adaptation and, thus, the fixation of a novel HA reassortant may occur in a human or perhaps an intermediate host such as quail [45], [46]. Overall, the required HA substitutions form a stringent barrier against the emergence of pandemic HA reassortants.

Taken together, at least three conceivable constraints on reassortment acting on different levels are conceivable: (I) putative strain-specific differences in vRNP packaging signals especially in the PB2, PA, NP, and M segments [47]; (II) disturbed indirect or direct interplay among the viral proteins such as an HA-NA imbalance of receptor affinity and enzymatic activity [48], [49], altered HA-NA cooperation [18], [50] or impaired PB2-NP interaction [24], [51], [52]; and (III) poor adaptation of virus proteins to the novel host environment such as inadequate HA receptor bias [37], [40], [43], [53]–[55] or reduced polymerase activity [25], [56], [57]. In this study, we obtained several avian HA reassortants which replicate comparably to the human parent virus. Therefore, even LPAIV like DkUkr63, which is poorly adapted to the human host, might transmit their HA to established human strains facilitating the emergence of novel strains with pandemic potential. Since the approach of this study is based on forced selection in-vitro (‘proof of principle’), the probability of such HA reassortants to emerge and to spread remains to be studied further in animal models. Since LPAIV with serotypes H1, H2 and H3, the only HA serotypes hitherto detected in established human strains, are frequently isolated from birds [10], those strains deserve sustained surveillance. Furthermore, a comprehensive distance matrix-based search among all publicly available influenza virus genomes for reassortant genotypes revealed a disproportionate high number of HA or HA/NA reassortants [58]. This finding supports the notion that both earlier (like Hk68 used in our study) and recent strains may form reassortants with avian strains although some human isolates may differ in that respect [18]. As the transmission of an avian virus HA into circulating human strains may not be restricted and the frequency of such reassortment events is unknown, LPAIV remain relevant for public health. Such a screening approach as used in this study may serve to identify easily transmissible HA genes from avian strains allowing to take them into account in vaccine planning. Overall, we suggest that the HA of LPAIV are easily transmissible to human viruses, however, the stable fixation of novel HA reassortants in the human population would require further adaptation of the HA gene including adequate receptor specificity.

## Materials and Methods

### Cells and Viruses

Human lung epithelium (A549), Madin-Darby kidney (MDCK) and human embryonic kidney (HEK293T) cells (cell lines 1035, 1064, and 1018, respectively, Collection of Cell Lines in Veterinary Medicine of the Friedrich-Loeffler-Institut) were maintained in minimal essential medium containing 10% fetal bovine serum. The human strain A/Hong Kong/1/68 (H3N2) (Hk68) and the avian strains A/Duck/Ukraine/1/63 (H3N8) (DkUkr63) (at 5^th^ egg passage) and A/Mallard/Germany/Wv64-67/2005 (H3N2) (MallGer05) (at 2^nd^ egg passage) were propagated in embryonated chicken eggs. The comparison of the Hk68 amino acid sequences to published sequences (accession numbers AF348170 (PB2), AF348172 (PB1), AF348174 (PA), AF348176 (HA), AF348180 (NP), AF348184 (NA), AF348188 (M), and AF348198 (NS)) yielded the following results: PB2 I584V, PB1 100% identity, PA N142T and K673R, HA 100% identity, NP Q311H, NA N339D, M1 100% identity, M2 100% identity, NS1 I81M, and NS2 100% identity.

### Generation of the Elastase-dependent Hk68-Ela

The HA gene of Hk68 (GenBank accession number AF348176; one silent nucleotide exchange at position 1668 A to G), cloned into plasmid pHWSccdB [13], was subjected to site-directed mutagenesis at the HA cleavage site by the Quikchange™ protocol (primer sequences available on request). By cotransfection of the mutated HA plasmid and the seven plasmids encoding the other genes of Hk68, we generated the mutant virus Hk68-Ela [59] and subsequently propagated and titrated it by plaque assay [60] on MDCK cells in the presence of 5.0 µg/ml porcine pancreatic elastase (Serva Electrophoresis GmbH).

### Co-infection Experiments for the Generation of HA Reassortants

A549 cells were co-infected with Hk68-Ela at a multiplicity of infection (MOI) of 1 and either DkUkr63 or MallGer05 (MOI of 10^−2^) in the absence of elastase, and either with (1.0 µg/ml) or without N-tosyl-L-phenylalanine chloromethyl ketone (TPCK)-treated trypsin (Sigma). After eight and 24 hours, we harvested the supernatants plaque-titrated them on MDCK cells in the presence of 2.0 µg/ml TPCK-treated trypsin. For each co-infection experiment, we picked 21 plaques and propagated them on A549 cells in the presence of 1.0 µg/ml trypsin. If no hemagglutination titer was detectable in the supernatants, we propagated the virus in 11-day-old embryonated chicken eggs.

### Growth Curves

For determination of growth curves, A549 cells were infected at a MOI of 10^−3^ and incubated in the presence of 1.0 µg/ml TPCK-treated trypsin. The supernatants from two or four independent experiments were harvested after 0, 8, 24, 48, and 72 h post inoculation and subjected to titration by plaque assay on MDCK cells in the presence of 2.0 µg/ml TPCK-treated trypsin. For pairwise comparison of the 48 h titers of different reassortants with those of the parent viruses, Wilcoxon tests were performed using R software [61], version 2.15.2 (2012-10-26). The exact conditional p-values for the Wilcoxon rank sum test [62] were calculated using the R-package ‘exactRankTests’ [63].

### Genotyping

RNA was extracted by using the RNeasy Kit (Qiagen). The viral RNA was then reverse-transcribed into cDNA using the Onestep Kit (Qiagen) together with a primer pair universal for NP, NA, M and NS genes [64] or primer pairs specific to the HA gene, modified from [65], or parts of the P gene segments. PCR products were cleaned after agarose gel electrophoresis using the Qiaquick gel extraction kit (Qiagen). Sequencing was performed by Seqlab Göttingen GmbH using internal primers to each of the 8 segments.

## Supporting Information

Figure S1 **Strict elastase dependency of Hk68-Ela.** The plaque assay was performed on MDCK cells in the presence of elastase or trypsin or in the absence of an exogenous protease.
(TIF)

Figure S2 **Plaque morphology of parental viruses and reassortants.** The plaque assays were performed on MDCK cells in the presence of trypsin except Hk68-Ela requiring elastase.
(TIF)

Table S1 (DOC)

Table S2 (DOC)

Table S3 (DOC)
