# Supplementary figures and images for: Avian Influenza Virus H3 Hemagglutinin May Enable High Fitness of Novel Human Virus Reassortants
Source: PLoS One. 2013 Nov 12;8(11):e79165. doi: 10.1371/journal.pone.0079165 (PMC3827155; doi:10.1371/journal.pone.0079165)

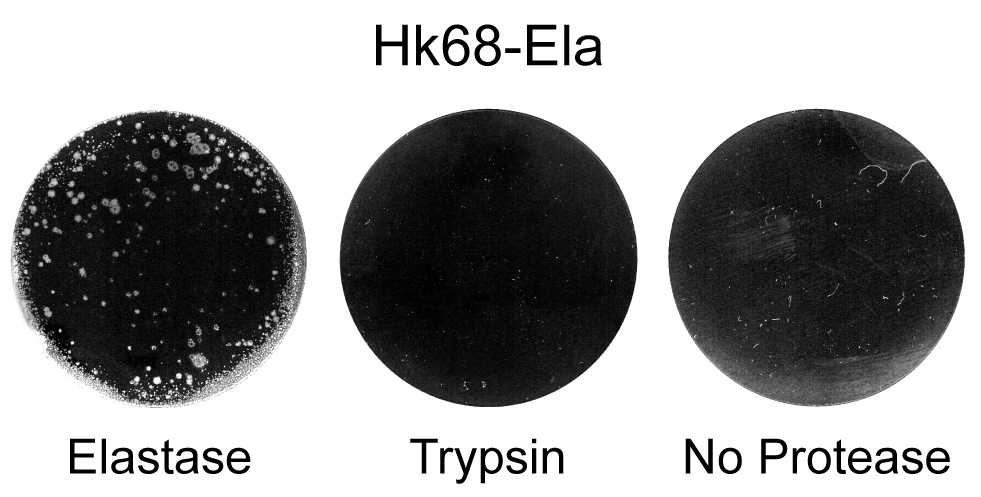

Supplement: Figure S1 — Strict elastase dependency of Hk68-Ela. The plaque assay was performed on MDCK cells in the presence of elastase or trypsin or in the absence of an exogenous protease. (TIF) [file pone.0079165.s001.tif]

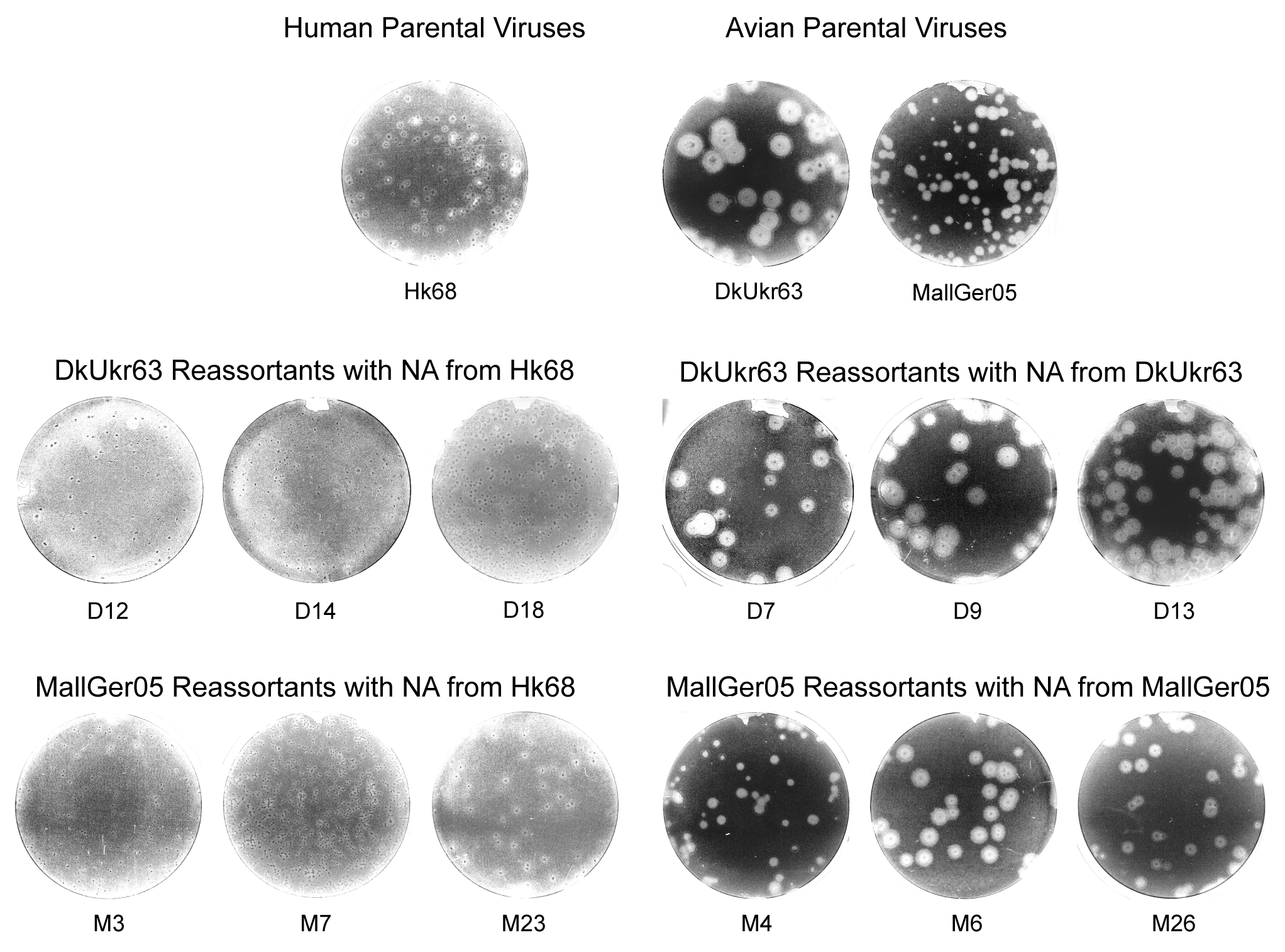

Supplement: Figure S2 — Plaque morphology of parental viruses and reassortants. The plaque assays were performed on MDCK cells in the presence of trypsin except Hk68-Ela requiring elastase. (TIF) [file pone.0079165.s002.tif]
