# Supplementary material for: Avian Influenza Virus H3 Hemagglutinin May Enable High Fitness of Novel Human Virus Reassortants
Source: PLoS One. 2013 Nov 12;8(11):e79165. doi: 10.1371/journal.pone.0079165 (PMC3827155; doi:10.1371/journal.pone.0079165)
Supplement: Table S2 — (DOC) [file pone.0079165.s004.doc]

**Table S2 Wilcoxon Rank Sum Tests of 48h Titers from the MallGer05 Reassortants versus Hk68 or MallGer05**

All average titers within one magnitude to that of Hk68 (> 7,8E+05) are printed in bold. The Hk68 and Hk68-Ela data are identical to those shown in Table S1. Abbreviations: Repl. – replicate, vs. – versus, n/a – not applicable, nd – not done, sign. – significant.
