# Supplementary material for: Avian Influenza Virus H3 Hemagglutinin May Enable High Fitness of Novel Human Virus Reassortants
Source: PLoS One. 2013 Nov 12;8(11):e79165. doi: 10.1371/journal.pone.0079165 (PMC3827155; doi:10.1371/journal.pone.0079165)
Supplement: Table S3 — (DOC) [file pone.0079165.s005.doc]

**Table S3 Number and gene compositions of reassortants growing comparable** to Hk68

| HA donor | DkUkr63 | MallGer05 |
| --- | --- | --- |
| Plaques  analyzed | 21 | 21 |
| Reassortant plaques | 16 | 18 |
| Estimated number of genotypes and confidence intervals | 15^1^/15^2^/(13,16)^2^ | 17^1^/17^2^(15,18)^2^ |
| Genotypes found | 7 | 14 |
| Genotypes found  with growth comparable to Hk68 | 4 | 10 |
| Gene Constellations  (avian virus genes) and Frequency of reassortants with growth comparable to Hk68 | HA: 5  (D21, D20, D11, D16, D12)  HA/NA: 4  (D3, D2, D10,D9)  HA/NP/NA: 2  (D1, D15)  PB1/HA/NA: 1  (D7) | HA/NA: 4  (M22, M26, M25, M21)  HA: 2  (M7, M35)  HA/NA/M: 1  (M36)  HA/NA/M/NS: 1  (M9)  HA/NP/M/NS: 1  (M23)  HA/M: 1  (M10)  PB1/HA/NA/M/NS: 1  (M6)  PA/HA/NP/NA/M: 1  (M1)  PA/HA/NP/NA/NS: 1  (M24)  PA/HA/NA/NS: 1  (M5) |

The numbers of all reassortant plaques are listed in comparison to the number of expected genotypes (i.e. unique gene combinations) calculated according to an exact formula derived from the coupon buyers problem ^1^ and Monte Carlo simulations (10.000 runs) together with their confidence intervals ^2^ and the genotypes established. Reassortants which reach titers not lower than one magnitude of the 48 h titer of Hk68 are considered to grow comparable to Hk68. From those reassortants, the gene constellation (avian virus genes) and their frequency are given.
